# Knowledge of Risk Factors for Dementia and Attitudes on a Dementia Prevention Program by Age and Ethnicity in Arizona

**DOI:** 10.14283/jarlife.2024.19

**Published:** 2024-12-16

**Authors:** H. Talkad, Y. Chen, A.P. Bress, J.B. Langbaum, P.N. Tariot, J.J. Pruzin

**Affiliations:** 1. University of Arizona College of Medicine - Phoenix, Phoenix, AZ, USA; 2. Banner Alzheimer’s Institute, Phoenix, AZ, USA; 3. University of Utah, Salt Lake City Utah

**Keywords:** Alzheimer’s disease, health equity, risk factor

## Abstract

**Background:**

Dementia disproportionately affects Hispanic communities, which may be partially attributable to disparities in resources to address modifiable risk factors. Addressing risk factors at younger ages would likely confer greater benefit than at older ages. Interest among Hispanic and younger persons participating in a dementia prevention program is unknown.

**Objectives:**

To understand knowledge of dementia risk factors and attitudes toward prevention program participation among Arizona residents.

**Design:**

Cross-sectional study.

**Setting:**

Online survey conducted of Arizona residents in either English or Spanish between July 13, 2021 and August 2, 2021.

**Participants:**

1,303 persons age 35 and older; 332 (25.5%) were Hispanic.

**Measurements:**

Survey questions about knowledge of dementia risk factors and dementia prevention program interest. Comparisons between Hispanic and non-Hispanic White and younger and older respondents were made using chi-squared tests.

**Results:**

Overall, 30.7% of respondents were aware of any risk factors that increased risk for dementia with no differences between Hispanic and non-Hispanic White respondents. 76.4% of all respondents were “very” or “somewhat” interested in a dementia prevention program, interest was significantly higher in Hispanic (83.0% vs 73.3% “very” or “somewhat interested,” X2 (3, N=1226) = 14.8, p=0.002) and younger respondents (82.2% vs 72.1% “very” or “somewhat” interested X2 (1, N=1302) = 20.0, p<0.001).

**Conclusion:**

General knowledge of risk factors for dementia is low, contrasting with high interest in a prevention program. Interest is higher in Hispanic and younger persons compared with older or non-Hispanic White persons. A dementia prevention program accessible to younger and Hispanic populations could help narrow dementia outcome disparities.

## Introduction

**A**lzheimer’s disease (AD), the most common cause of dementia, results from a long and complex pathophysiological process that begins at least two decades before the appearance of symptoms (1). While the accumulation of beta-amyloid (Aβ) plaques followed by hyperphosphorylated tau-containing neurofibrillary tangles define AD and are likely largely responsible for symptoms, the degree to which, and precisely how the presence of plaque and tangle pathology influence cognitive and functional symptoms remain incompletely understood. Many additional factors significantly influence the absolute risk and age of onset of both AD and AD-related dementias (AD/ADRD), many of which are modifiable (2). Previous studies highlight the importance of these risk factors, estimating that about 40% of dementia cases are attributable to them, and thus potentially preventable if fully addressed (2). Physical activity slows cognitive decline (3, 4) and lowers risk for AD/ADRD (5, 6). Sleep disturbances in middle age increase risk of dementia (7, 8). Loneliness increases risk, while strong social engagement is protective (9). Although recent evidence is mixed, eating a Mediterranean diet may lower risk (10, 11). Avoiding smoking (12), moderating alcohol consumption, treating hearing loss, obesity, and depression, and avoiding traumatic brain injury and polluted air also reduce the risk of developing dementia later in life (2, 10).

While risk factor modification in older adults is important in addressing AD/ADRD, epidemiological evidence shows that midlife may represent a crucial window to address many risk factors. For example, for diabetes and hypertension, the longer one has the condition and the more poorly controlled, the greater the later life dementia risk (13-15). Early intervention on modifiable risk factors will likely be more effective in reducing dementia risk compared to interventions at ages more proximate to a typical dementia diagnosis.

Next, interventions accessible to minoritized communities could help narrow AD/ADRD outcome disparities. Unaddressed modifiable risk factors and inequities in social determinants of health likely play an important role in driving disparities. Black and Hispanic persons are 1.5-2 times more likely than non-Hispanic persons to develop AD/ADRD (16). They have less brain Aβ (17) but more cerebral small vessel disease (18), indicative of differences in the etiology of AD/ADRD among ethnicities, likely reflecting the degree to which conditions like hypertension and diabetes are adequately treated (14, 15, 19). Additionally, risk factors like diet and physical activity level vary in relationship to race and socioeconomic status, which may be due to differences in the built environment, including access to parks and quality food, and neighborhood walkability (20, 21).

A small number of cognitive health clinics aimed at providing care and recommendations to delay or even prevent the onset of dementia have been established to address AD/ADRD through optimization of modifiable risk factors, with early evidence suggestive of a benefit (22, 23). However, the focus of these programs is often on older individuals and does not emphasize including marginalized populations. While dementia prevention programs may provide the most benefit to younger persons and those in disadvantaged communities, the degree of interest in cognitive health clinics aimed at dementia prevention in general and attitudes about participation in such programs among younger persons and those in marginalized Hispanic communities are unknown. To address this knowledge gap, we surveyed Arizona residents age 35 and over about knowledge of modifiable risk factors for dementia and attitudes toward prevention programs to address risk factors. We next examined differences in responses between Hispanic and non-Hispanic White respondents as well as between younger and older respondents (age 35-54 vs 55 and over) to determine the interest and feasibility of establishing a cognitive health clinic to reduce dementia risk serving middle-aged residents, with an emphasis on Hispanic community participation, in Arizona.

## Methods

### Survey Data Collection

We partnered with SSRS, a professional market and survey research firm, to survey Arizona residents age 35 or older using an online self-administered questionnaire in either English or Spanish between July 13, 2021 and August 2, 2021. The questions were developed as a practical, exploratory approach to gather information about knowledge of AD/ADRD risk factors and attitudes toward participation in dementia prevention and research. The questions of interest are shown in Table 1. The study utilized third-party opt-in web unpaid panels with potential respondents being invited via email. Within the survey, respondents were screened to ensure that they met qualification criteria. Multiple panels were used to ensure adequate representation of hard-to-reach respondents, including Hispanic and Black households as well as those with income <$25,000 per year. Extensive program checks were conducted to ensure that skip patterns followed the design of the questionnaire, and the program was tested via desktop computer as well as mobile devices to ensure consistent visualization across devices prior to execution. The final program used various quality checks, including questions asking respondents to select a specific answer to be sure they were reading each question, a “low incidence item” question and a “speeder trap” to ensure the final sample included only high-quality surveys.

**Table 1.** Survey Questions

1. Has a close friend or relative of yours ever been diagnosed with Alzheimer’s disease or dementia? Yes or No
2. How worried are you about a close relative being diagnosed with Alzheimer’s disease or dementia in the future? Very worried, Somewhat worried, Not too worried, Not worried at all
3 How worried are you about being diagnosed with Alzheimer’s disease or dementia in the future? Very worried, Somewhat worried, Not too worried, Not worried at all
4. Are you aware of any risk factors that increase the risk for developing Alzheimer’s disease or dementia later in life? Yes or No
5. Of the following factors listed below, please indicate which are risk factors for Alzheimer’s disease or dementia. -High blood pressure -Diabetes -Depression -Poor sleep quality -Hearing loss -Eating an unhealthy diet -Not being physically active -Lack of social interaction Is a risk factor or is not a risk factor
6. How interested would you be in participating in a program focused on minimizing the chances of developing Alzheimer’s disease or dementia in the future? Very interested, Somewhat interest, Not too interested, Not at all interested
7. Being a participant in the program would allow our team to know you well as a person and patient. If a drug or clinical trial to prevent, delay, or treat Alzheimer’s disease or dementia became available, the Comprehensive Dementia Prevention Community program could refer participants for the possibility of participating in the trial. How interested would you be in this type of referral? Very interested, Somewhat interest, Not too interested, Not at all interested

The survey sample sought to mirror the demographic composition of Arizona, the geographic area of interest. We employed weighting to compensate for sample designs and patterns of non-response that might bias results. To handle missing data among some of the self-reported demographic variables for purposes of weighting only, we employed a technique termed “hot decking,” which replaces the missing values of a respondent randomly with another similar respondent without missing data. These are further determined by variables predictive of non-response that are present in the entire file (see technical appendix for more details) (24, 25). Weighting was accomplished using SPSS INC RAKE, an SPSS extension module that simultaneously balances the distributions of all variables. The weighting parameters were sex, age, education, race/ethnicity, region of Arizona, civic engagement, marital status, and household income, derived from the September 2019 Current Population Survey Volunteering and Civic Life Supplement (civic engagement) or the 2019 American Community Survey (sex, age, education, race/ethnicity, region of Arizona, marital status, and household income). Weights were trimmed at the 2nd and 98th percentiles to prevent individual interviews from having too much influence on the final results.

### Statistical Analysis

All data analyses were performed in SPSS version 27. We calculated descriptive statistics for survey questions of interest in the whole group, then by ethnicity and by age group for questions of interest. We then compared the distribution of answers between Hispanic and non-Hispanic White respondents at all ages with additional comparisons made between the ethnic groups in only those at younger ages, between 35-54 years-old, using chi-squared tests. We also examined differences in responses between all participants by age, comparing younger respondents (age 35-54) to older respondents (55 and over), again using chi-squared tests.

## Results

A total of 1,303 persons aged 35 and older, of whom 332 (25.5%) were Hispanic and 842 (64.6%) non-Hispanic White, responded to the survey between July 13th and August 2nd, 2021. 63.3% of respondents were female. The characteristics of the survey population are shown in Table 2.

**Table 2.** Respondent Characteristics

	**All**	**Non-Hispanic White**	**Hispanic**
Age			
All, mean (SD)	57.7 (13.2) n=1225	62.0 (11.9) n=842	48.2 (11.0) n=383
35-54 years, mean (SD)	44.0 (5.8) n=500	45.6 (6.0) n=215	42.8 (5.3) n=285
55+ years, mean (SD)	67.2 (7.3) n=725	67.6 (7.2) n=627	64.0 (7.3) n=98
Sex			
Male, No. (%)	443 (36.2%)	284 (33.7%)	159 (41.5%)
Female, No. (%)	778 (63.5%)	555 (65.9%)	223 (58.2%)
Other, No. (%)	4 (0.3%)	3 (0.4%)	1 (0.3%)
County			
Urban (Maricopa or Pima)	881 (71.9%)	615 (73.0%)	266 (69.5%)
Rural (Other)	344 (28.1%)	227 (27.0%)	117 (30.5%)
Income			
<$50k, No. (%)	450 (36.7%)	309 (36.7%)	141 (36.8%)
>$50k, No. (%)	589 (48.1%)	380 (45.1%)	209 (54.6%)
Blank, No. (%)	186 (15.2%)	153 (18.2%)	33 (8.6%)
Education			
<High School, No. (%)	40 (3.3%)	15 (1.8%)	25 (6.5%)
>High School, No. (%)	1185 (96.7%)	827 (98.2%)	358 (93.5%)

### Attitudes in Hispanic Compared to Non-Hispanic White Respondents

Hispanic respondents were more likely to report having a close friend or relative who had been diagnosed with AD or dementia compared to non-Hispanic White respondents (66.8% vs 51.9%, X2 (1, N=1226) = 24.1, p<0.001). Hispanic respondents also reported a higher degree of concern about a close relative (73.1% vs 63.2% being “very” or “somewhat worried”, X2 (3, N=1226) = 40.3, p<0.001) as well as themselves (71.3% vs 55.6% being “very” or “somewhat worried”, X2 (3, N=1226) = 63.1, p<0.001) being diagnosed with AD or dementia in the future compared to Non-Hispanic White respondents. The concern about a future diagnosis being more prominent among Hispanic respondents remained evident when limiting analyses to younger individuals 35-54 years old (75.4% vs 69.8% “very” or “somewhat interested,” X2 (3, N=500) = 16.5, p<0.001).

**Figure 1.**
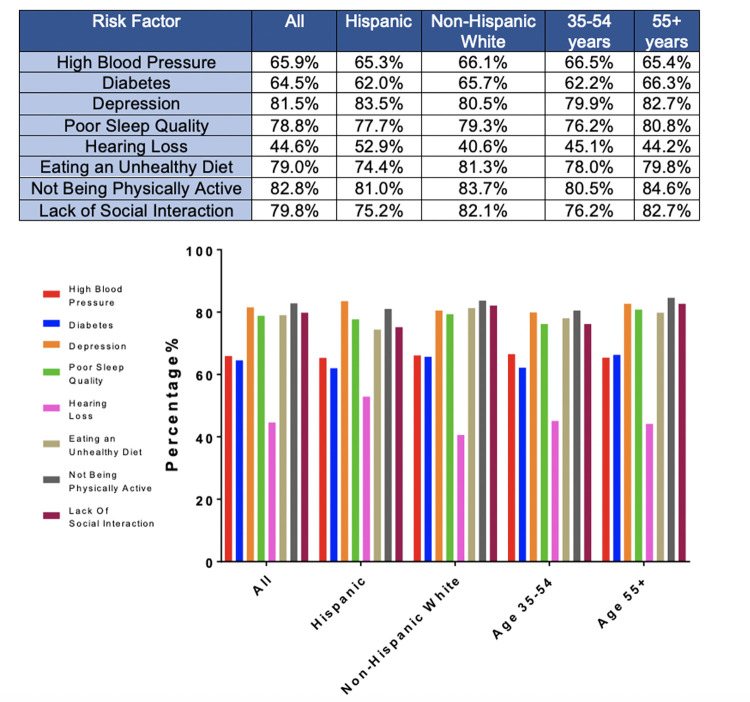
Awareness of risk factors by demographic group

When asked, “are you aware of any risk factors that increase the risk for developing AD or dementia later in life?” (Table 1, Question 4), 30.7% of respondents reported being aware of such factors and there was no significant difference between non-Hispanic White and Hispanic respondents (31.6% vs 29.8%, X2 (1, N=1226) = 0.41, p=0.52). When the general question about risk factors was followed up by asking whether a specific item such as diabetes or physical inactivity are risk factors for dementia (Table 1, Question 5), the percentage of persons reporting awareness of individual risk factors increased significantly, generally doubling or more with the exception of hearing loss. Hearing loss was the risk factor with the least awareness, with Hispanic respondents being more likely to be aware of this risk factor. There were no statistically significant differences between the two groups in awareness of any other risk factors. Figure 1 summarizes the percentages of persons reporting awareness of individual risk factors for AD/ADRD in the overall group, as well as by ethnicity and age.

**Figure 2.**
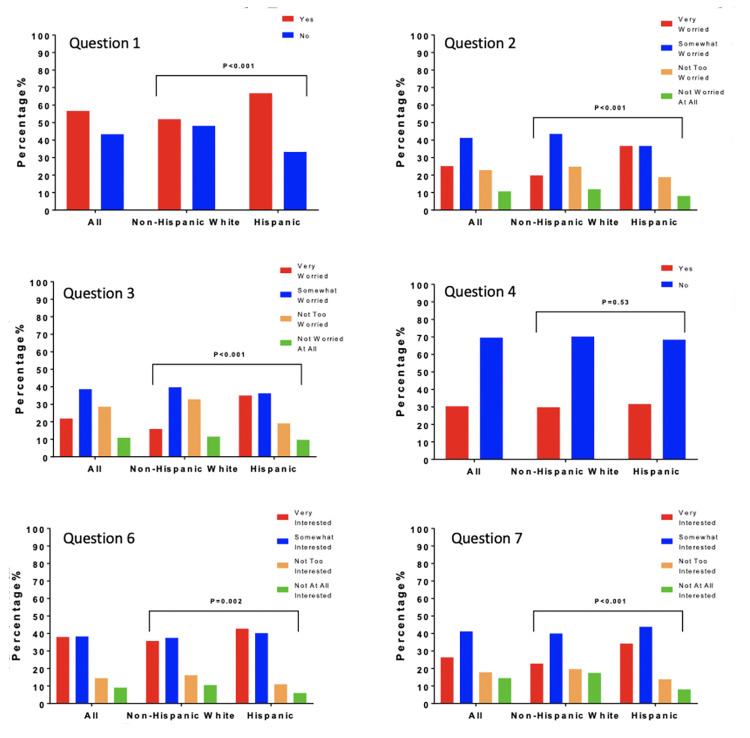
Distribution and Comparison of Responses of Hispanic and Non-Hispanic White Participants

We next asked about interest in a dementia prevention program (Table 1, Question 6). 76.4% of respondents reported being “very” or “somewhat interested” in such a program. Interest was significantly higher in Hispanic compared to non-Hispanic White respondents (83.0% vs 73.3% “very” or “somewhat interested,” X2 (3, N=1226) = 14.8, p=0.002). Reponses of the entire sample and comparisons between Hispanic and non-Hispanic White respondents for this and other survey questions are summarized in Figure 2. Hispanic male respondents were significantly more likely than non-Hispanic White male respondents to have interest in a dementia prevention program (86.2% vs. 71.9% being “very” or “somewhat interested,” X2 (3, N=444) = 15.2, p=0.002), while Hispanic female respondents were numerically more interested in a program compared to non-Hispanic White females, the difference in interest was not significant (80.7% vs. 74.1%, X2 (3, N=778) = 3.9, p=0.269). Stratifying by income level did not significantly alter results, Hispanic respondents expressed greater interest in a dementia prevention program in both the low (<$50,000/year) and greater (>$50,000/year) income groups (83.0% vs. 71.9% being “somewhat” or “very interested” for the income <$50,000 group, X2 (3, N=450) = 8.0, p=0.046, and 84.7% vs. 71.4% X2 (3, N=590) = 13.2, p=0.004 for the income >$50,000 group). Hispanic participants remained more interested in a prevention program regardless of education level (X2 (3, N=40) = 12.9, p=0.005 for those with a high school degree or less, X2 (3, N=1186) = 12.6, p=0.005) for those with more than a high school degree).

Next, we asked about interest in participation in a clinical trial aimed at preventing or delaying Alzheimer’s disease or dementia (Table 1, Question 7). Hispanic respondents had greater interest in participating in AD/ADRD prevention research (78.1% Hispanic vs 62.8% Non-Hispanic White were “very” or “somewhat interested”, X2 (3, N=1226) = 35.1, p<0.001). This finding was consistent when limiting analyses to younger respondents, age 35-54 (80.7% vs. 68.4%, X2 (3, N=500) = 15.6, p=0.001) as well as both only male (82.4% vs. 61.8%, X2 (3, N=444) = 30.3, p<0.001) and only female respondents (74.9% vs. 63.1%, X2 (3, N=778) = 10.4, p=0.015).

### Attitudes in Younger Compared to Older Respondents

**Figure 3.**
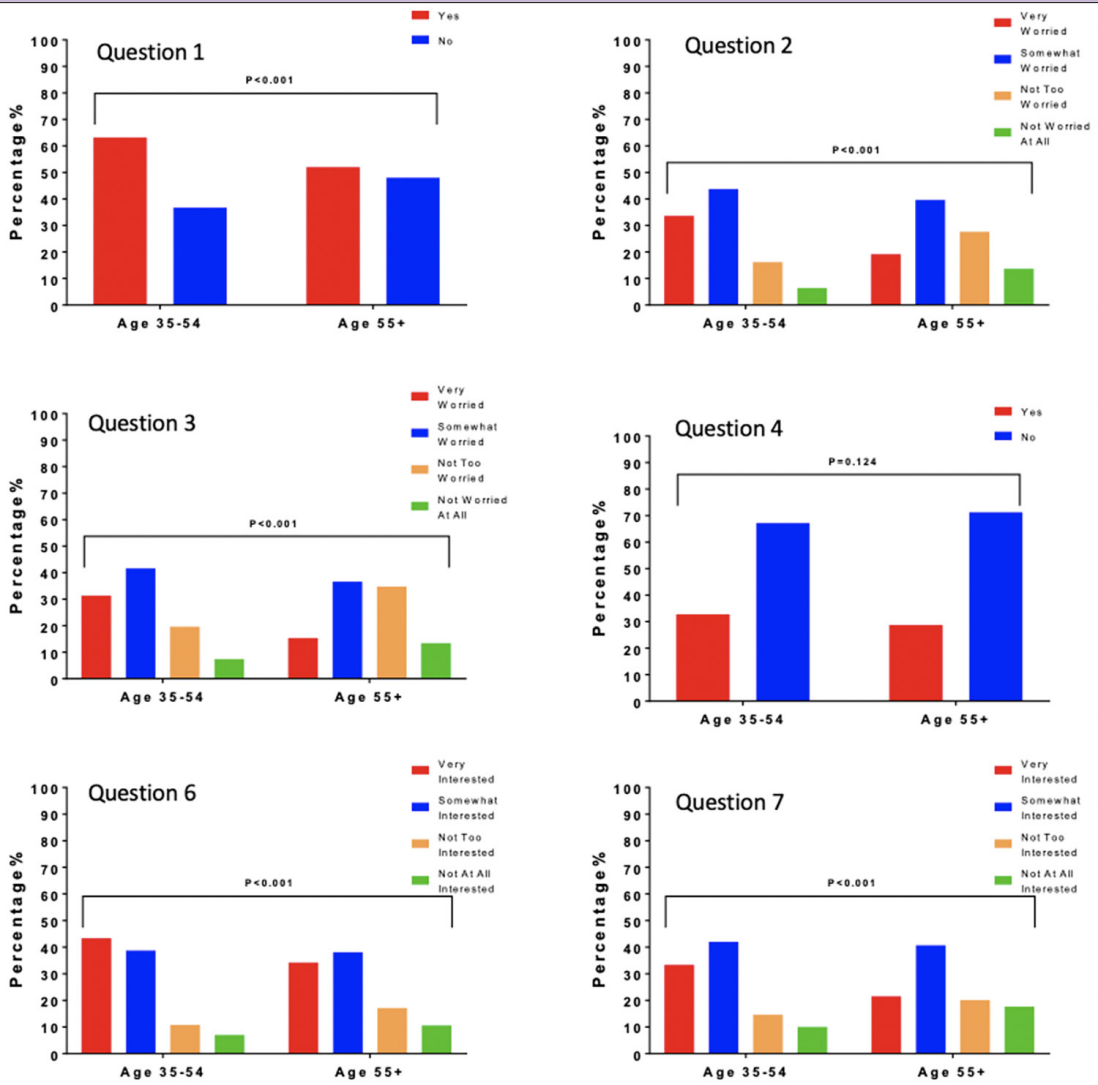
Distribution and Comparison of Responses of Participants Ages 35-54 Compared to Those 55+

Younger participants (age 35-54) were more worried about themselves being diagnosed with AD or another dementia in the future (72.4% vs. 51.5%, X2 (3, N=1302) = 74.9, p <0.001) compared to older respondents (age 55 and older). This remained the case when comparing younger with older Hispanic respondents, with 74.8% of the younger group versus 61.1% of the older group reporting that they were either “very” or “somewhat worried” (X2 (3, N=383) = 9.9, p=0.019). Comparisons between younger and older respondents are shown in Figure 3.

There were no significant differences between younger and older persons in awareness of risk factors that increase the risk of developing AD/ADRD later in life (33.4% vs. 28.8%, X2 (1, N=1302) = 3.1, p =0.076) though again surprisingly the trend was for younger persons to be more aware of risk factors. Interest in participating in a program targeting dementia prevention was greater in the younger population. 83.0% of younger participants were either “very interested” or “somewhat interested” in participating in a dementia prevention program versus 72.1% of older participants (X2 (3, N=1302) = 20.0, p<0.001). Younger respondents also reported significantly greater interest in being referred for a drug or clinical trial to prevent, delay, or treat AD or dementia if one were available (75.0% vs. 61.7% being “somewhat” or “very interested,” X2 (3, N=1302) = 33.6, p<0.001).

## Discussion

This study provides insight into the degree of awareness of risk factors for dementia, interest in participating in a prevention program, and respective differences between Hispanic and non-Hispanic White persons as well as younger and older persons in Arizona. We found that a significant majority of respondents reported not knowing about any risk factors for dementia, with no difference between Hispanic and non-Hispanic White participants. While less than 1/3 of respondents reported being aware of risk factors, about 3/4 were interested in dementia prevention programs, indicating that an important disconnect exists between the desire for addressing modifiable risk factors to lower dementia risk and the ability to accomplish this goal.

Next, we found that Hispanic respondents had a higher degree of interest in a dementia prevention program than non-Hispanic White respondents. This may follow from the fact that Hispanic respondents were also more likely to know someone with dementia and be more worried both about a relative and themselves developing dementia in the future. This interest, paired with the fact that Hispanic persons have more prevalent and poorly addressed modifiable risk factors, suggests dementia prevention programs have the potential to provide greater benefit to Hispanic communities, which could narrow outcome disparities. It is important to point out that, at a minimum, access to such clinics would need to be equitable, if not somewhat designed, tailored, and with recruitment skewed toward underrepresented communities. While interest in a dementia program is encouraging, interest alone does nothing to address existing barriers to implementation (28). Increasing healthcare resources, both in terms of education and delivery, are required to meet the existing interest in underrepresented communities to make an impact. Increasing investment in preventative and early intervention programs for common but addressable risk factors for dementia may be one such strategy.

This study also found that Hispanic respondents were more interested in participating in a prevention clinical trial than non-Hispanic White respondents. This contrasts with historical underrepresentation of Hispanic participants in AD/ADRD clinical trials and suggests that the problem is not a fear of, or a lack of interest in participation, but rather efforts of studies and trials to reach and provide access to diverse participants. Congruent with our finding is a national survey that found no differences in willingness to participate in a prevention trial among socioeconomic groups (26) and the recent report from the United States Study to Protect Brain Health Through Lifestyle Intervention to Reduce Risk (US POINTER), which successfully enrolled 30.8% non-White participants (27).

Most clinical programs and trials are focused on older individuals. Here, we found interest in a dementia prevention program was higher among younger individuals ages 35-54 compared to those age 55 and older suggesting that inclusion of younger individuals in prevention clinics is feasible. There may be several reasons for this finding. Cognitive decline and dementia were considered to be a normal part of aging in previous decades. Concerted efforts have sought to dispel this notion, and younger individuals may acknowledge the fact that dementia is not a normal part of aging more than older individuals. They may also be aware of interventions to lower the risk of this outcome, whereas older individuals might view cognitive decline and dementia as an inevitable part of aging. Another possible explanation is in the changing attitudes regarding overall health among generations, with prioritization of “wellness,” (defined as “the active pursuit of activities, choices, and lifestyles that lead to a state of holistic health” (28)) being more prominent in younger generations. For example, with respect to diet, millennials (those born between 1981 and 1996) are much more likely (80% vs 64%) to prioritize health and wellness when making food choices as compared to baby boomers (those born between 1946 and 1964) (29). A similar phenomenon may be occurring in this study, with younger persons having a greater interest in proactively maintaining their health and wellness through lifestyle choices. Finally, there may be some degree of selection bias in the sample, with younger persons being more likely to participate in the survey if they had prior experience and knowledge of dementia and interest in ways to lower future risk.

### Limitations

Study limitations include a reliance on self-reported data on education, income, and other demographic data. The study was conducted online and thus likely excluded some persons with no or little access to reliable internet. The sample was predominately female (63.3%). Next, respondents were not paid to complete the survey, and there may be some degree of selection bias of persons who have an interest in dementia prevention and modifiable risk factors as being more likely to offer their time and effort to complete the survey. This selection bias may have been introduced to a higher degree in younger persons who would presumably be more occupied with their careers and younger families, requiring a greater interest in the topic to motivate participation and completion. These limitations may have rendered the survey population less representative of the general population, reducing generalizability, and potentially skewing results to some degree. The sample was also intentionally representative of Arizona residents, and thus may not be generalizable beyond this geographic area. These limitations could be mitigated in future research by increasing geographic reach, obtaining more complete demographic information, offering monetary compensation for participation, and including non-web-based/electronic formats to promote participation among those with limited internet access.

## Conclusions

Among a representative sample of Arizona residents, knowledge of modifiable risk factors for dementia was low, contrasting with the high degree of interest in a dementia prevention program addressing these risk factors. Interest in a clinic was higher among Hispanic compared to non-Hispanic White and younger compared to older respondents. Implementation of a cognitive health clinic aimed at dementia prevention inclusive of young and diverse participants is feasible in Arizona. Provided that access is equitable among ethnic groups, a dementia prevention program addressing modifiable risk factors may be one way to narrow AD/ADRD outcome disparities.

## Supplemental Materials

Additional material Supplementary document file supplied by authors.

### Technical Appendix

We provide additional details about how “hot-decking” was used for purposes of weighting for this survey data. In general, hot deck imputation can be a practical solution to missing data problems, and a computational tool exists for this technique in SPSS.^1^ Hot deck imputation replaces a respondent’s missing data with that of another similar respondent without missing data. Respondents are considered similar if their data are identical for a selection of variables determined by the researcher. This selection of variables is related to the data being imputed, predictive of non-response, and are present in the entire file. Collectively, these variables are referred to as the “deck.” After segmenting respondents into groups based on the values of the deck, all respondents within each group are randomly sorted and respondents with missing data are assigned the data of the respondent nearest to them who is not missing data. This process is equivalent to assigning values to nonresponses by randomly sampling, without replacement, from the distribution of values from respondents with the same set of values on the deck variables as the respondent.^2^

In the specific case of this survey, data were imputed for weighting only, and only for gender. There were five cases that identified as “other” gender. Since the Census does not allow for a non-binary gender option, gender was imputed to these five cases for weight purposes only. In the data reported, there are no imputed variables (hot-deck or otherwise). Whether values were assigned (for weighting purposes only) to these handful of cases using hot-deck imputation (or any other method) or not, it would not make a difference on the actual data and results of analyses, especially for the minimal amount the technique was used (5 respondents who listed gender as “other”).

1Myers
T.
Goodbye, Listwise Deletion: Presenting Hot Deck Imputation as an Easy and Effective Tool for Handling Missing Data. Communication Methods and Measures. 2011;5:297-310. doi: 10.1080/19312458.2011.624490.2Andridge
RR, Little
RJ. A Review of Hot Deck Imputation for Survey Non-response. Int Stat Rev.
2010;78(1):40-64. doi: 10.1111/j.1751-5823.2010.00103.x. PubMed PMID: 21743766; PMCID: PMC3130338.21743766
PMC3130338
