# Supplementary material for: Knowledge of Risk Factors for Dementia and Attitudes on a Dementia Prevention Program by Age and Ethnicity in Arizona
Source: JAR Life. 2024 Dec 16;13:126–34. doi: 10.14283/jarlife.2024.19 (PMC11653765; doi:10.14283/jarlife.2024.19)
Supplement: Additional material — Supplementary document file supplied by authors. [file jarlife-13-0019-S1.docx]

Technical Appendix

We provide additional details about how “hot-decking” was used for purposes of weighting for this survey data. In general, hot deck imputation can be a practical solution to missing data problems, and a computational tool exists for this technique in SPSS.^1^ Hot deck imputation replaces a respondent’s missing data with that of another similar respondent without missing data. Respondents are considered similar if their data are identical for a selection of variables determined by the researcher. This selection of variables is related to the data being imputed, predictive of non-response, and are present in the entire file. Collectively, these variables are referred to as the “deck.” After segmenting respondents into groups based on the values of the deck, all respondents within each group are randomly sorted and respondents with missing data are assigned the data of the respondent nearest to them who is not missing data. This process is equivalent to assigning values to nonresponses by randomly sampling, without replacement, from the distribution of values from respondents with the same set of values on the deck variables as the respondent.^2^

In the specific case of this survey, data were imputed for weighting only, and only for gender. There were five cases that identified as “other” gender. Since the Census does not allow for a non-binary gender option, gender was imputed to these five cases for weight purposes only. In the data reported, there are no imputed variables (hot-deck or otherwise). Whether values were assigned (for weighting purposes only) to these handful of cases using hot-deck imputation (or any other method) or not, it would not make a difference on the actual data and results of analyses, especially for the minimal amount the technique was used (5 respondents who listed gender as “other”).

1. Myers T. Goodbye, Listwise Deletion: Presenting Hot Deck Imputation as an Easy and Effective Tool for Handling Missing Data. Communication Methods and Measures. 2011;5:297-310. doi: 10.1080/19312458.2011.624490.

2. Andridge RR, Little RJ. A Review of Hot Deck Imputation for Survey Non-response. Int Stat Rev. 2010;78(1):40-64. doi: 10.1111/j.1751-5823.2010.00103.x. PubMed PMID: 21743766; PMCID: PMC3130338.
